# Socioeconomic, Ethnic, Racial, and Gender Gaps in Children’s Social/Behavioral Skills: Do They Grow Faster in School or out?

**DOI:** 10.15195/v6.a17

**Published:** 2019-05-29

**Authors:** Douglas B. Downey, Joseph Workman, Paul von Hippel

**Affiliations:** a Department of Sociology, The Ohio State University.; b Department of Sociology, University of Missouri-Kansas City.; c Lyndon B. Johnson School of Public Affairs, The University of Texas at Austin.

**Keywords:** schools, inequality, children, social/behavioral skills

## Abstract

Children’s social and behavioral skills vary considerably by socioeconomic status (SES), race and/or ethnicity, and gender, yet it is unclear to what degree these differences are due to school or nonschool factors. We observe how gaps in social and behavioral skills change during school and nonschool (summer) periods from the start of kindergarten entry until the end of second grade in a recent and nationally representative sample of more than 16,000 children (the Early Childhood Longitudinal Study Kindergarten Class of 2010–11). We find that large gaps in social and behavioral skills exist at the start of kindergarten entry, and these gaps favor high-SES, white, and female children. Over the next three years, we observed that the gaps grow no faster when school is in than when school is out. In the case of social and behavioral skills, it appears that schools neither exacerbate inequality nor reduce it.

MORE than 50 years after the landmark 1966 Coleman Report, there is still little scholarly consensus regarding how schools shape inequality (Downey and Condron 2016). The surprising conclusion from the Coleman Report was that schools do not seem to influence achievement gaps in math and reading skills much, a position prompting an energetic response from education scholars who contended that disparities in school quality across race and socioeconomic status (SES) are highly consequential. Recently, this debate has been dominated by analyses from seasonal-comparison scholars who observe how gaps in math and reading test scores change when school is in versus out. The majority of this scholarship has mostly confirmed Coleman’s original conclusion that schools play a modest role in shaping achievement gaps (Downey, von Hippel, and Broh 2004; Quinn et. al. 2016; von Hippel, Workman, and Downey 2018; von Hippel and Hamrock 2019).^1^

An important concern with this more positive portrayal of schools, however, is that seasonal analyses have primarily been limited to analyzing how schools affect children’s math and reading skills. Although promoting children’s cognitive skills is an important school function, schools could shape children’s lifelong trajectories in other ways that are independent of cognitive skills (Carter 2016). It would be premature, therefore, to temper the critical view of schools based on the current seasonal evidence because that view has yet to consider schools’ broader impact on children’s lives.

We test whether the more favorable view of schools emerging from seasonal-comparison studies extends to children’s social and behavioral skills, operationalized as good citizenship in the classroom. Good classroom citizenship is characterized by meeting the teacher’s expectations along a number of dimensions, such as paying attention, following directions, trying hard to complete work, getting along with other students, avoiding disruptive behavior, and exhibiting self-control. We focus on social and behavioral skills for three reasons: (1) Although they correlate with math and reading skills, they are distinct (Jennings and DiPrete 2010); (2) they are important for success in life, and some would argue they are even more important than cognitive skills (Brunello and Schlotter 2011; Deming 2017; Heckman, Stixrud, and Urzua 2006); and (3) there exist seasonally collected observations of these skills.

We observe the size of gaps when children start school and how gaps change when school is in session and when school is out for summer vacation. This seasonal-comparison approach has several advantages. First, it recognizes that children arrive at kindergarten with large gaps in social and behavioral skills already in place. This method is less vulnerable, therefore, to misattributing gaps formed in early childhood to school factors. Second, it circumvents the formidable methodological challenge of identifying and measuring all of the school mechanisms at stake. We apply the seasonal-comparison method to social and behavioral skills with nationally representative data spanning kindergarten, first grade, and second grade (three school years and two summers).

## Do Schools Exacerbate Inequality in Social and Behavioral Skills?

It is well established that low-SES, black, and male children exhibit poorer social and behavioral skills than their counterparts (DiPrete and Jennings 2012), but it is difficult to know schools’ role in this relationship. One reason to expect the gaps to increase is that disadvantaged children may endure poorer teachers. Teachers vary in quality when it comes to promoting math and reading skills, and it turns out that they vary even more in how well they promote children’s social and behavioral skills. Jennings and DiPrete (2010) analyzed kindergartners in the Early Childhood Longitudinal Study Kindergarten Class of 1998–99 and concluded that if a student enjoyed an above-average (rather than below-average) kindergarten teacher, they experienced a 0.042–standard deviation (SD) boost in math and a 0.141–standard deviation improvement in reading. Comparatively, children’s social and behavioral skills increased by 0.185 standard deviations if exposed to an above-average rather than a below-average teacher.^2^ If disadvantaged children are exposed to teachers less skilled at nurturing the development of social and behavioral skills, we would expect gaps in these skills to increase during school periods.

Although the teacher may be the dominant factor, peers may also shape gaps in social and behavioral skills. Black and low-SES students more frequently attend schools with disadvantaged peers who may have low levels of social and behavioral skills themselves. These peers may disrupt instruction, require additional teacher attention, and undermine the developmental environment. The notion that one’s peers play an important role in shaping children’s school experiences has a long history with respect to academic performance (Hanushek et al. 2003; Sacerdote 2001). For example, Hoxby (2002) found that being surrounded by peers who score one SD higher on an assessment of academic skills results in raising a student’s own score by between 0.15 and 0.40 SDs. It is plausible that the benefits of exposure to advantaged peers (and the costs of exposure to disadvantaged ones) extend to social and behavioral skills.

In addition, the regimented nature of the school day may be disproportionately challenging for some groups of students, especially those who arrive at kindergarten with less developed citizenship skills. For children already behind on these skills, the school environment may largely serve to accentuate existing disparities in citizenship. And as children progress through school, teachers’ expectations for children to sit still, pay attention, and focus on their academics likely increase, potentially leading to growing gaps in social and behavioral skills among social groups.

## Or Do Schools Reduce Inequality?

**Figure 1:**
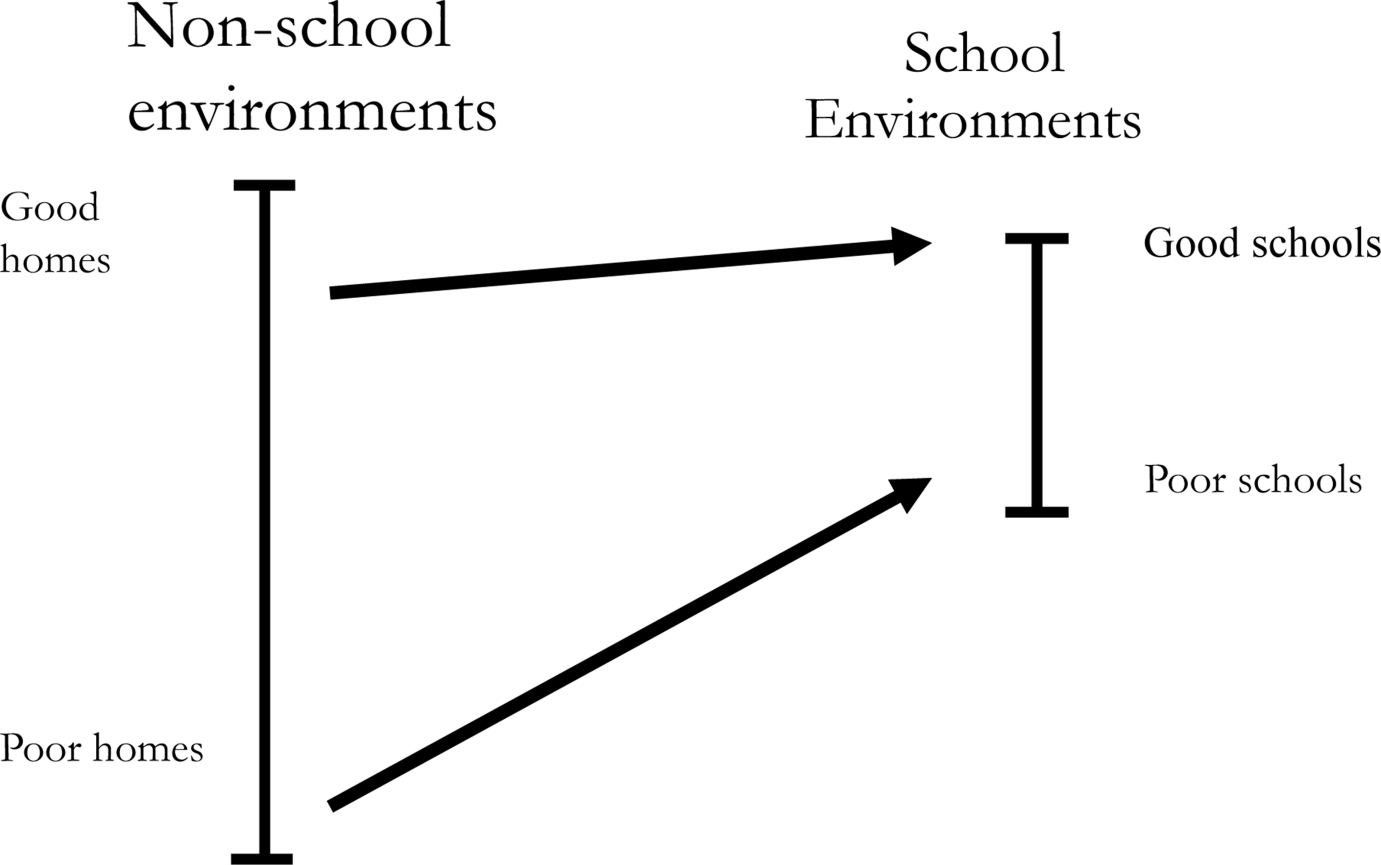
A contextual view: variation in nonschool and school environments. Adapted from Downey et al. (2004).

Although there are significant reasons for believing that schools might exacerbate gaps in social and behavioral skills, we are sensitive to the possibility that schools may play little role or may even reduce gaps. One way this could happen is if schools are more equal environments (than nonschool environments) for children’s social and behavioral development (Figure 1).

From this perspective, schools may provide a neutral ground or could even favor privileged children. However, whatever preferential environments advantaged children enjoy at school would be more modest than those enjoyed at home. In this way, unequal schools could still be an equalizing force simply because they are a more equal environment than widely unequal nonschool environments.

Second, it is possible that teachers target the improvement of social and behavioral growth of disadvantaged children. Low-SES and black children arrive at kindergarten with weaker social and behavioral skills (DiPrete and Jennings 2012), and so it could be that teachers provide greater attention to children who struggle to fit the “student role.” There is some evidence that teachers do this with respect to cognitive skills. For example, a national survey of teachers found that, when asked who was most likely to receive one-on-one attention, 80 percent of teachers said “academically struggling students,” whereas just 5 percent said “academically advanced” students (Duffett, Farkas, and Loveless 2008). Perhaps teachers also devote disproportionate attention to students who are struggling with social and behavioral skills, and school programs may also target the improvement of children most at risk. If so, these school mechanisms could be a force for reducing gaps in social/behavioral skills across social groups.

Discerning between these two perspectives—schools reduce versus increase gaps—is especially challenging with traditional methodological approaches for two reasons. First, most scholarship studying social and behavioral skills fails to gauge the magnitude of gaps at the onset of schooling in kindergarten or first grade. This turns out to be important when studying cognitive skills, as scholars are learning that gaps in skills across socioeconomic status, race, and gender are mostly formed before schools have a chance to matter (von Hippel and Hamrock 2019; von Hippel et al. 2018). If gaps in social and behavioral skills are similarly formed mostly during early childhood, then scholarship may produce a distorted view of schools’ role by overlooking this pattern. Second, it is problematic to assess how schools matter with traditional methods that rely on statistically controlling for indicators of children’s nonschool environments because it is so difficult to identify and measure perfectly all relevant nonschool factors. We refer to this approach as “covariate adjustment” and note that it is vulnerable to overestimating schools’ role and underestimating the importance of the nonschool environment. Walberg (1984) reminds us that the average 18-year-old American has spent just 13 percent of their waking hours in school. The nonschool environment, therefore, represents a hefty confounder when attempting to isolate school effects.

## A Seasonal-Comparison Approach to Social and Behavioral Skills

These challenges prompt us to approach the question from an “exposure” framework and a seasonal-comparison research design. The analytic approach is simple: We ask, “Do gaps in skills grow more when children are exposed to school than when they are not?” We leverage the seasonal nature of the American school calendar—nine months of school followed by a three-month summer break—which provides a natural experiment for understanding how schools matter (Gangl 2010). Previous scholars have employed this research design to compare how gaps in math and reading (Downey et al. 2004; Entwisle and Alexander 1992; Heyns 1978; Quinn et al. 2016; von Hippel and Hamrock 2019; von Hippel et al. 2018) and gaps in body mass index (von Hippel et al. 2007; von Hippel and Workman 2016) change when school is in versus out.

The seasonal-comparison research design has produced a largely favorable picture of how schools influence inequality. For example, many scholars have found evidence that socioeconomic gaps in reading skills grow similarly when school is out versus in or sometimes even faster when school is out (Downey et al. 2004; Entwisle and Alexander 1992; Quinn et al. 2016; von Hippel et al. 2018; von Hippel and Hamrock 2019), a pattern prompting Alexander (1997) to conclude that, when it comes to inequality, “schools are more ‘part of the solution’ than ‘part of the problem”‘ (16) and for Downey et. al. (2004) to conclude that schools really are a “great equalizer.”

Although the patterns that emerge from seasonal-comparison studies of cognitive skills are encouraging, some scholars have pointed out that schools might be compensatory with respect to cognitive skills but increase inequality in other ways (Carter 2016). Expanding the seasonal method beyond cognitive skills, therefore, is necessary to create a more comprehensive understanding of schools’ influence on inequality. We note that cognitive and social/behavioral skills are mutually constitutive and reinforcing (DeAngelis 2018; DiPrete and Jennings 2012; Owens 2016). Children who self-regulate and can readily pay attention, for example, are also more likely to learn more reading and math skills. And children who learn more reading and math skills are likely better positioned to self-regulate and pay attention. We would expect, therefore, to observe that schools shape cognitive and social/behavioral skills in a similar manner, but we note the possibility that schools influence these distinct skills in unique ways.

## Social and Behavioral Skills: The Gap at Kindergarten Entry and How It Changes by Fifth Grade

Some of the best evidence for the size of gaps in social and behavioral skills at kindergarten entry comes from DiPrete and Jennings’s (2012) analysis of the nationally representative Early Childhood Longitudinal Study Kindergarten Class of 1998–99. They developed a composite of social and behavioral skills and then estimated gaps across socioeconomic status (poor vs. nonpoor), race, and gender. Their composite measure included teacher evaluations of children’s approaches to learning (attentiveness, task persistence, eagerness to learn, learning independence, flexibility, and organization), self-control (ability to control behavior by respecting the property rights of others, controlling temper, accepting peer ideas for group activities, and responding appropriately to pressure from peers), and interpersonal skills (skill in forming and maintaining friendships; getting along with people who are different; comforting or helping other children; expressing feelings, ideas, and opinions in positive ways; and showing sensitivity to the feelings of others).

DiPrete and Jennings (2012) found that the vast majority of the fifth-grade gaps in social/behavioral skills were already in place at kindergarten entry. For example, the authors note that 86 percent of the poor-nonpoor gap at fifth grade was already in place at kindergarten entry. Similarly, white students were far ahead of black students at fifth grade, but 87 percent of this gap was in place at kindergarten entry. And finally, girls were rated as having better social/behavioral skills than boys in fifth grade, but 79 percent of this gap was in place at kindergarten entry. Although these percentages should not be viewed as definitive because the social/behavioral scales are not interval scaled, even so, we can say that most of the “action” generating inequality in social and behavioral skills occurs in early childhood, before schools have a chance to matter.

What do these patterns mean for the role of schools? Although the gaps only grow modestly between kindergarten and fifth grade, they at least grow some, which is an indication that schools might have played a role in increasing them. But it is hard to know based on this evidence. For example, the black-white gap at kindergarten entry was shaped by many home and neighborhood factors that likely continued to matter over the next six years. Even if schools played a neutral role, therefore, we would expect the black-white gap in fifth grade to be larger than the kindergarten gap. Indeed, it is even possible that the black-white gap grew between kindergarten and fifth grade in spite of schools.

These issues lead us to address this main research question: Do gaps in social/behavioral skills grow faster when school is in versus out?

## Methods

### Sample

We analyzed data from the Early Childhood Longitudinal Study Kindergarten (ECLS-K) Class of 2010–11, a nationally representative probability sample of children who were followed from the fall of kindergarten in 2010 through the spring of fifth grade in 2016. The ECLS-K employed a multistage clustered sampling design: It first sampled primary sampling units (PSUs), each of which consisted of a single large county or a group of similar and adjacent small counties. Within each PSU, it sampled schools, and within each school, it sampled students. Eighteen percent of children had missing values on at least one covariate: 10 percent were missing age, 5 percent were missing SES, and 2 percent were missing both. Race and/or ethnicity and gender were missing for less than 1 percent of children. After dropping children with missing covariates and no social/behavioral measures in any round, our analytic sample consisted of 14,029 students sampled from 836 schools. We used sampling weights to compensate for oversampling and nonresponse.^3^

We focused on the first three years of study, when children’s social and behavioral skills were measured twice per year: in the fall and spring of kindergarten, first grade, and second grade. These twice-yearly measurements permitted us to estimate growth in social and behavioral skills separately for each of the first three school years and each of the first two summer vacations. Measurements in the fall of first and second grade were limited to a subsample containing one-third of the sampled PSUs. Subsampling reduced power but did not introduce bias because the subsample was taken at random. We included all children with any measures on the dependent variable in our analyses because our random effects longitudinal models use full information maximum likelihood to model all observations of the dependent variable and did not require that every child be observed on every occasion.

### Dependent Variables

Teachers evaluated children’s social and behavior skills along five dimensions defined by the ECLS-K (Tourangeau et al. 2017). The scale for each dimension was constructed by summing four to six questionnaire items. Responses on each item ranged from never (1) to very often (4). Sums were standardized by round for some analyses. The dimensions and component items were as follows:

1. Approaches to learning was a sum of questions asking whether the child kept belongings organized, (b) showed eagerness to learn new things, (c) worked independently, (d) adapted easily to changes in routine, (e) persisted in completing tasks, (f) paid attention well, and (g) followed classroom rules. The reliability of the Approaches to Learning Scale, according to the internal consistency of the component items (Cronbach’s alpha), was 0.91 on average and varied little by round (see Tables 3–9 in Tourangeau et. al [2017], reproduced in Appendix A in our online supplement).
2. Self-control was a sum of questions evaluating the child’s ability to (a) control behavior by respecting the property rights of others, (b) control temper, (c) accept peer ideas for group activities, and (d) respond appropriately to pressure from peers. The average reliability of the Self-Control Scale was 0.81.
3. Interpersonal skills was a sum of questions evaluating the child’s ability to (a) form and maintain friendships; (b) get along with people who are different; (c) comfort or help other children; (d) express feelings, ideas, and opinions in a positive way; and (e) show sensitivity to the feelings of others. The average reliability of the Interpersonal Skills scale was 0.86.
4. Internalizing problem behaviors was a sum of questions asking how often the child (a) felt angry when having trouble learning, (b) worried about taking tests, (c) felt lonely, or (d) worried about doing well in school. The reliability of this scale was 0.79.
5. Externalizing problem behaviors was a sum of questions asking how often the child (a) argued, (b) fought, (c) got angry, (d) acted impulsively, or (e) disturbed ongoing activities. The reliability of this scale was 0.88.

All measures of social/behavioral skills were coded so that higher scores indicated more favorable behavior. To do this, we reverse coded externalizing problem behaviors and internalizing problem behaviors. Although additional measures of social/behavioral skills were available in some rounds (e.g., the Student-Teacher Relationship Scale), these were only assessed in the spring of kindergarten and first grade, so we could not include them in our seasonal analyses.

Subjective teacher evaluations likely varied across teachers with different standards for behavior. This subjectivity presented a challenge for assessing children’s growth over time, especially summer growth because children are rated by different teachers before and after summer vacation. Some teachers may have evaluated children more leniently on average than others. This problem is not unique to our study but is endemic to the literature on social and behavioral skills, which commonly relies on reports by teachers or parents, neither of whom are impartial.

Two issues are worth noting with respect to teachers’ subjective evaluations. First, although teacher evaluations might simply reflect the values of the dominant cultural group, the proportion of variance that lies between schools is very small for social and behavioral skills (about 8 percent), a pattern that Jennings and DiPrete (2010) note “strongly suggests that teachers within schools rate students on social and behavioral skills in comparison with students similar to their within-school peers (e.g., previous students that the teacher has instructed) rather than with the broader population of students across the country” (148). Second, some scholarship has attempted to assess whether evaluations of minority students represent teacher bias or real behavioral differences. For example, disproportionate numbers of black and Hispanic students are referred into disability and special-education categories, highlighting the possibility that teachers unfairly rate minority students. But Hosterman, DuPaul, and Jitendra (2008) compared teacher evaluations (used for referral) with classroom observations made by an independent observer and concluded that teacher ratings of minority students accurately reflected children’s behaviors.

Although there is no way to entirely eliminate subjectivity from the scales, we partly addressed the challenge by estimating models with teacher fixed effects. Teacher fixed effects control for constant or average differences between the use of the scales by different teachers, but they do not control for the possibility that teachers apply the scale differently to different students in their classrooms. For example, if teacher A is more lenient than teacher B and scores all students about a point higher than teacher B would, teacher fixed effects will adjust for that. But if teacher A is only lenient with white students, whereas teacher B is equally strict with students of all races, then this difference will not be controlled by teacher fixed effects—or any other method.

A second challenge is that the social and behavioral measures may not be interval scales. A one-point difference at the bottom of the scale may not be comparable to a one-point change at the top. This is especially a problem for research on gap growth, which necessarily compares students who start at different points on the scale. Again, this problem is not unique to our study but is endemic to the literature on growth in social and behavioral skill gaps. Although there is no way to transform the measures to a scale that is guaranteed to have the interval property, we tested the robustness of our conclusions by standardizing scores using the mean and SD at each round of the survey (fall kindergarten, spring kindergarten, etc.). Even though there is no guarantee that the standardized scores had the interval property either, by comparing results before and after standardization, we could assess the degree to which the results were sensitive to one simple transformation.

Although our scales are necessarily imperfect, it is helpful that we measure students on five different scales. Unless each scale suffers from exactly the same problems (which seems unlikely), confidence in our results will increase to the degree that we observe similar results across different scales.

### Independent Variables

Parents reported their children’s race and/or ethnicity, gender, and age at kindergarten entry. In addition, the ECLS-K 2011 included a measure of family SES constructed by summing standardized measures of parents’ reports of years of education, household income, and occupation, which the ECLS-K converted to an occupational prestige score. In our tables, we report comparisons between the top and bottom SES quintiles. We focus on black-white gaps because previous seasonal-comparison scholarship has found evidence that schools exacerbate black-white gaps in math and reading.

### Analytic Strategy

Our data allow us to compare how gaps change during school periods (kindergarten, first grade, and second grade) with how they change during nonschool periods (the summers after kindergarten and first grade). This analytic approach requires assumptions to produce unbiased causal estimates of the effect of schools. First, we assume that there was no spillover between treatment and nontreatment periods—that is, that what happened during the school year did not affect growth in social or behavioral skills during the summer. This assumption could be violated if, for example, a child’s school experience was so traumatic that it influenced both their end-of-year rating and their development over the summer. Second, we must assume that, other than school exposure, nothing else that affected social and behavioral skills varied across seasons. We could produce biased estimates if, for example, children’s social and behavioral skills were influenced by temperature change and different social groups of interest experienced these changes differently. As a contrived example, imagine that boys were more subdued by warm temperatures than girls, so boys’ behavior improved more during the summers and worsened more during the winters. This scenario, although not highly plausible, would bias our estimates, making school exposure for boys appear worse than it really was.

### Model

We analyzed school-year and summer growth in social and behavioral–skill gaps using a two-level random-intercept model (Raudenbush and Bryk 2002). For child i in school j, various social/behavioral skills $Y_{ijt}$ were measured on six different occasions. The occasions were near the beginning and end of kindergarten, first grade, and second grade. On each occasion, the child had been exposed to a certain number of months of kindergarten ($G0_{ijt}$), first grade ($G1_{ijt}$), and second grade ($G2_{ijt}$) and a certain number of summer months after kindergarten ($S0_{ijt}$) and after first grade ($S1_{ijt}$). Children varied in the amount of school and summer exposure that they had at each measurement occasion. On average, children started kindergarten on August 25 and were first measured on October 10, so at the time of the first measurement, they had approximately $G0_{ijt}=1.5$ months of summer exposure, but this varied across schools. The mean and SD of school start dates, social and behavioral assessment dates, and school end dates appear in the online supplement as Table A1.

Our growth model adjusted for variation in school and summer exposure within and between children. At level 1, the model regressed the outcome on the exposure variables:

(1) $$Y_{ijt}=\alpha_{0}+\alpha_{1ij}G0_{ijt}+\alpha_{2ij}S0_{ijt}+\alpha_{3ij}G1_{ijt}+\alpha_{4ij}S1_{ijt}+\alpha_{5ij}G2_{ijt}+u_{i}+r_{j}+e_{ijt}.$$

Here, $\alpha_{0}$ was the average Y level on the first day of kindergarten, $u_{i}$ and $r_{j}$ were child- and school-level random effects representing departures from the average,^4^ $\alpha_{1ij}$ through $\alpha_{5ij}$ were child-specific growth rates during each school year and summer, $\alpha_{1ij}$ was the growth of Y per month of kindergarten, $\alpha_{2ij}$ was the growth of Y per month of the summer after kindergarten, and so on. The last term $e_{ijt}$ was residual error. A variant of the model included a fixed effect for each teacher.

At levels 2 and 3, we let the growth parameters $\alpha_{ij}=\left[\begin{array}{c} \alpha_{0ij}\ldots \alpha_{5ij} \end{array}\right]$ vary according to child characteristics. Let $X_{1ij},X_{2ij},\ldots .$ be a set of dummy variables indicating a child’s race, ethnicity, gender, and SES quintile. Gender was coded as 1 for girls and 0 for boys. Race and ethnicity were coded with dummies for black, Hispanic, Asian, Native American, and mixed-race children, with non-Hispanic white children being the omitted category. SES was coded with two dummy variables— one for the top SES quintile and one for the middle three quintiles—with the bottom quintile being the reference category.

Then, the growth parameters depended on the X variables as follows:

(2) $$\left[\begin{array}{l} \alpha_{0ij}\\ \alpha_{1ij}\\ \alpha_{2ij}\\ \alpha_{3ij}\\ \alpha_{4ij}\\ \alpha_{5ij} \end{array}\right]=\left[\begin{array}{l} \gamma_{00}\\ \gamma_{01}\\ \gamma_{02}\\ \gamma_{03}\\ \gamma_{04}\\ \gamma_{05} \end{array}\right]+\left[\begin{array}{l} \gamma_{10}\\ \gamma_{11}\\ \gamma_{12}\\ \gamma_{13}\\ \gamma_{14}\\ \gamma_{15} \end{array}\right]X_{1ij}+\left[\begin{array}{l} \gamma_{20}\\ \gamma_{21}\\ \gamma_{22}\\ \gamma_{23}\\ \gamma_{24}\\ \gamma_{25} \end{array}\right]X_{2ij}+\ldots .$$

For example, if $X_{1ij}$ is 1 for girls and 0 for boys, then during kindergarten, $\gamma_{01}$ was the monthly average growth rate for boys, and $\gamma_{11}$ was the monthly average growth in the gap between girls and boys. Likewise, during summer 1, $\gamma_{02}$ was the monthly average growth rate for boys, and $\gamma_{21}$ was the monthly growth in the gap between girls and boys. A similar interpretation applied to the other comparisons. Note that variation that was not associated with the X variables was modeled by the residual and random effects in Equation (1). Stata code to fit the model is available in Appendix B in the online supplement.

## Results

### Descriptive Patterns

At kindergarten entry, there were already large gaps in social and behavioral skills (Table 1). For example, between children in the highest and lowest SES quintiles, the standardized gap in approaches to learning was 0.49 SDs (0.32 for highest SES quintile vs. –0.17 for bottom quintile; *p <* 0.001), the standardized white-black gap is 0.27 SDs (0.15 for white vs. –0.12 for black students; *p <* 0.001), and the standardized girl-boy gap is 0.42 SDs (0.29 for girls vs. –0.13 for boys; *p <* 0.001). These gaps were comparable to those reported by previous scholars (Reardon and Portilla 2016).

**Figure 2:**
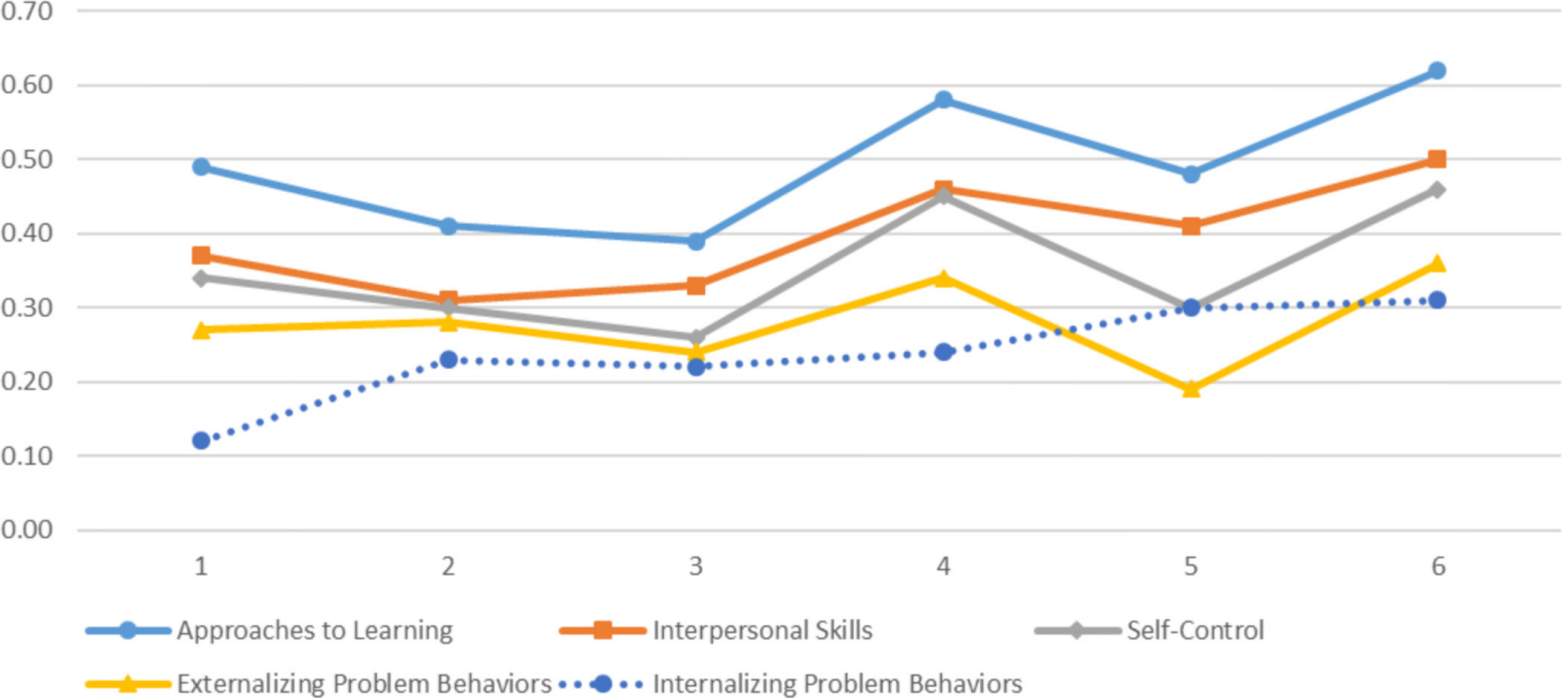
SES gap in social and behavioral skills.

**Figure 3:**
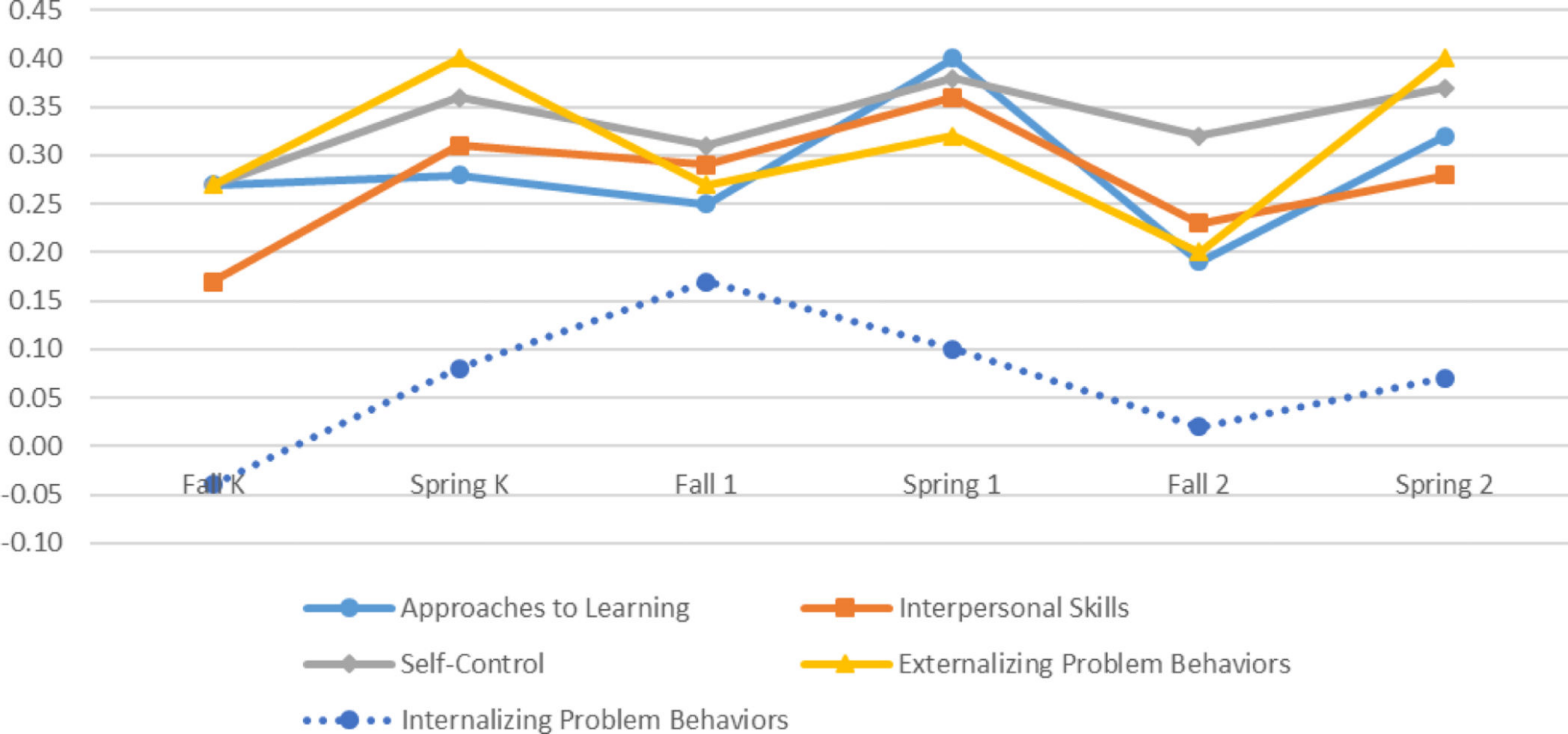
White-black gap in social and behavioral skills.

**Figure 4:**
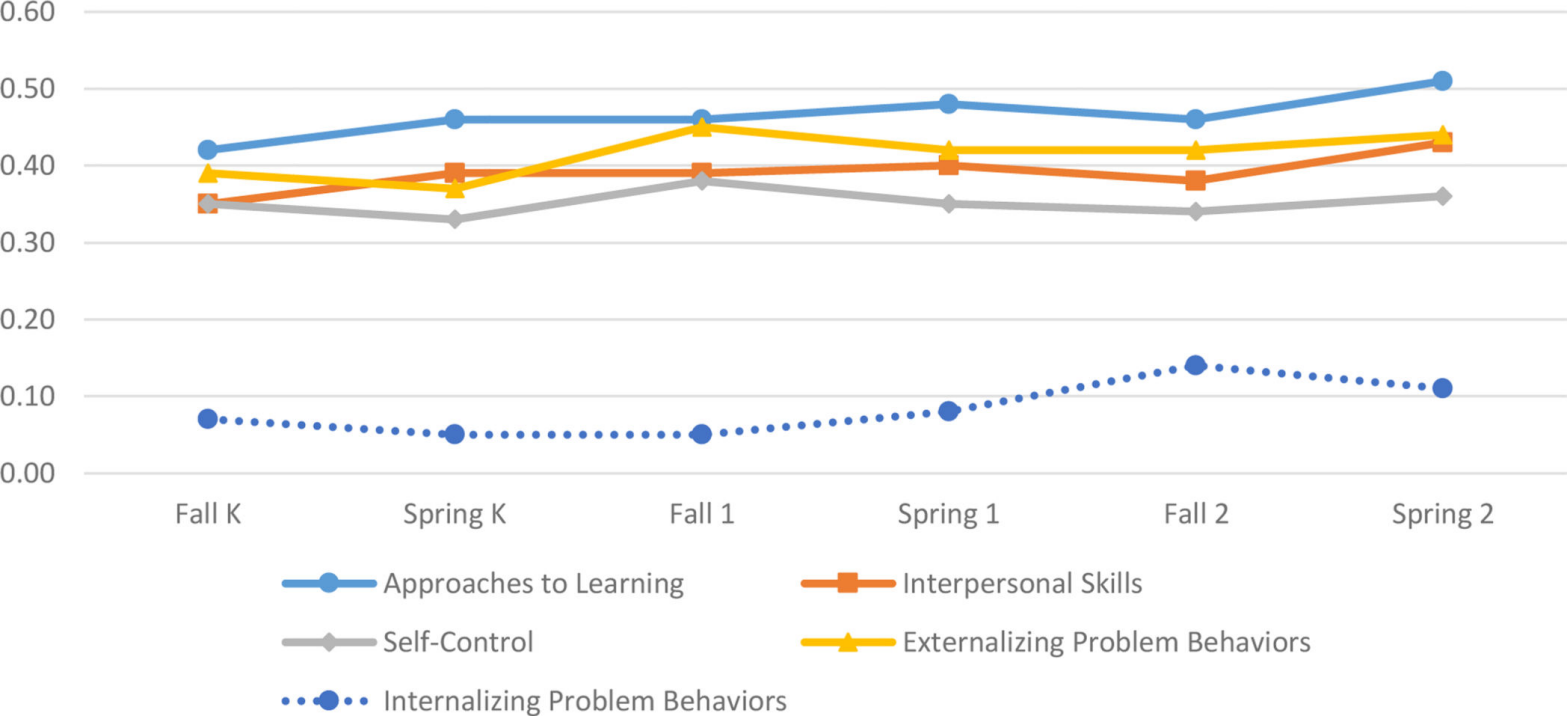
Female-male gap in social and behavioral skills.

As DiPrete and Jennings (2012) observed in the 1999 cohort of the ECLS-K, in the 2011 cohort, we found that gaps at kindergarten entry increased by about one-fifth by the end of second grade. In approaches to learning, for example, the standardized SES gap increased from 0.49 to 0.62 SDs (21 percent), the black-white gap increased from 0.27 to 0.32 SDs (16 percent), and the girl-boy gap increased from 0.42 to 0.51 SDs (18 percent). About 80 percent to 85 percent of the SES, race, and gender gaps in social and behavioral skills were in place at kindergarten entry. See Figures 2, 3, and 4 for graphic displays of changes in all social and behavioral gaps between the fall of kindergarten entry and the spring of second grade.

### Do Gaps Grow Faster When School Is in versus out?

The descriptive results confirm that gaps grew after school starts, but they do not tell us whether gaps grew more when school was in session or when school was out for summer vacation. Our multilevel growth models addressed that question. Table 2 presents the coefficients of the models predicting approaches to learning, with and without teacher fixed effects for both unstandardized and standardized scores. Results for the other four dependent variables, which are largely similar, appear in Appendix C in the online supplement.

Gaps in approaches to learning at kindergarten entry, which were observed in simple descriptive statistics (Table 1), are also evident in these multilevel models (Table 2). For example, in the standardized model without teacher fixed effects, girls arrived at kindergarten 0.464 SDs ahead of boys, black children arrived 0.130 SDs behind white children of the same SES, and top-quintile-SES children arrived 0.469 SDs ahead of bottom-quintile-SES children of the same race (Table 2, column 3). Estimated race and SES gaps are somewhat larger when we include teacher fixed effects, which compare students who have the same teacher. Specifically, if we add teacher fixed effects, then among children with the same teacher, girls started 0.461 SDs ahead of boys, black children started 0.251 SDs behind white children of the same SES, and children from high-SES families started 0.506 SDs ahead of children from low-SES families of the same race (Table 2, column 7). All our models include school random effects, which produce gap estimates that are a weighted average of within- and between-school differences (Greene 2011; Wooldridge 2001), and do not necessarily agree with the simple average differences in Table 1. These differences notwithstanding, all the models agree that there were substantial gaps, especially by gender and SES, at the start of kindergarten.

How did these gaps change after children start school? The coefficients in Table 2 estimate how the gaps changed over the next three years: during kindergarten, the first summer vacation, first grade, the second summer vacation, and second grade. There are very few statistically significant coefficients, and none are consistently significant across models and school years. For example, according to the two models without teacher fixed effects, black children appeared to lose statistically significant ground to white children during kindergarten and second grade, but these losses become nonsignificant in the models with teacher fixed effects. Likewise, in the models without teacher fixed effects, the gap between high- and low-SES children appeared to shrink during kindergarten, grow during first grade, and display no change during second grade, but most of these SES coefficients became nonsignificant when the model added teacher fixed effects. And finally, in the unstandardized model without fixed effects, girls appear to gain relative to boys during second grade (coefficient 0.006), but this pattern does not reach statistical significance in the other three models. Considering Table 2 overall and the number of coefficients at stake, the dominant pattern is that the growth of gaps in approaches to learning was not concentrated either in the school year or summer.

To develop a more comprehensive answer to the question of whether gaps grew faster during summer or during school, we tested whether the average of the school-year coefficients (kindergarten, first grade, and second grade) differed significantly from the average of the two summer coefficients. We summarize these contrasts in Table 3 for all five social and behavioral skills (not just approaches to learning) across the three social group comparisons (female-male, white-black, and high vs. low SES) and across our four different models (unstandardized and standardized dependent variable with and without teacher fixed effects). For example, in approaches to learning, the female-male contrast in the first model (a nonsignificant estimate of 0.0097) represents the average of the three school-year coefficients minus the average of the two summers. In other words, it asks whether the gap between girls and boys grows more during the school periods or summers. The lack of statistical significance for this contrast indicates that the gap between girls and boys grows no faster when school is in than when it is out (or vice versa).

The vast majority of contrasts do not reach statistical significance. There are a few hints of gaps growing at different rates during school and nonschool periods, but these results do not replicate across different dependent variables or model specifications. For example, black children appear to lose relative to white children during school periods with respect to approaches to learning in models that do not include teacher fixed effects, but the difference does not maintain statistical significance in models that include teacher fixed effects. Similarly, high-SES children gain relative to low-SES children while in school on the externalizing problem behaviors outcome in models without teacher fixed effects but not in models with teacher fixed effects. Finally, girls gain on boys while in school in models with teacher fixed effects predicting internalizing problem behavior but not in models without fixed effects.

These few statistically significant contrasts are overwhelmed by the dominant pattern: Only six of the 54 contrasts in Table 3 are statistically significant at *p <* 0.05, and only seven are significant at *p <* 0.10. And in no case do we find evidence that a gap grows faster during school versus nonschool periods across all four models. Before interpreting the handful of significant coefficients, we should remember that the standard *p <* 0.05 (or *p <* 0.10) means that, if the null hypothesis were true, we would expect 5 percent (or 10 percent) of estimates to reach statistical significance by chance. That is, if the null hypothesis were true, six of the 60 contrasts would be significant at *p <* 0.10, and three would be significant at *p <* 0.05. In fact, we observe only slightly more significant contrasts than would be expected under the null; seven contrasts are significant at *p <* 0.10, and six are significant at *p <* 0.05. If we applied a Bonferroni correction for multiple tests, none of these contrasts would reach statistical significance. We conclude that our results provide little reason to reject the null hypothesis that gaps in social and behavioral skills grow at the same rate during school and during summer—to the degree that gaps grow at all.

## Discussion

Determining schools’ role in the stratification system is challenging because children’s development is a product of both school and nonschool environments. We addressed this challenge by employing a seasonal design to observe how gaps change when children are in school versus out. Our main news is that exposure to schools does not appear to play a consistent role in either increasing or decreasing gaps in social and behavioral skills across socioeconomic status, racial and ethnic groups, or gender during the first three years of formal schooling. SES and gender gaps are large when children start school, and racial gaps are smaller (when SES is controlled); and none of these gaps changes much during the first three school years or the first two summer vacations.

The null results of our study are important. Our study had substantial statistical power, and our sample was large and nationally representative. Notably, results were consistent across different scales and different ways of analyzing them, suggesting that our patterns are not just a function of measurement artifacts, which presumably would be specific to certain scales. We showed large gaps at kindergarten entry, consistent with past studies and bolstering confidence that our measures and methods could detect gaps when gaps are present. In short, if schools increase gaps in social and behavioral skills, our study was well positioned to detect it.

Although this is the first study to apply seasonal-comparison methods to social and behavioral skills, the same approach has previously been used for reading and math skills and for obesity. Each time, seasonal comparisons have produced findings suggesting that schools’ overall effect on inequality is either neutral or compensatory (Downey et al. 2004; Entwisle and Alexander 1992; Quinn et al. 2016; von Hippel et al. 2007, 2018; von Hippel and Hamrock 2019; von Hippel and Workman 2016). And each time, seasonal-comparison studies direct our attention more toward nonschool environments as the major source of inequalities.

Our sample was limited to young children, from kindergarten until the end of second grade, and so our conclusions are restricted to the early stages of formal schooling. It is possible that schools play a more substantial role in increasing gaps in social and behavioral skills among older children. During middle and high school, for example, it may be that tracking mechanisms that result in greater within-school separation among advantaged and disadvantaged children throughout the day would aggravate gaps in social and behavioral skills. We caution, however, that this is speculation, and one can readily generate ideas in the opposite direction, too. For example, we might expect schools to become more compensatory as children move through the school system because multiple elementary schools typically feed into the same middle school, and multiple middle schools often feed into the same high school, increasing disadvantaged children’s exposure to advantaged peers (Fiel and Zhang 2018) and reducing between-school variation. Until seasonal research is carried out in secondary school, we are reluctant to make strong claims about how middle and high schools influence gaps in social and behavioral skills.

Although there may be school mechanisms that exacerbate inequality in social and behavioral skills, our results suggest that they are countered by others that reduce inequality. The seasonal research design may address the broad structural question of what role the school system, on average, plays in the stratification system. With respect to social and behavioral skills, that role appears to be neutral.

## Supplementary Material

Supp Material

## Figures and Tables

**Table 1:** Average social and behavioral skills by socioeconomic status, race (black or white), and gender from the start of kindergarten through the end of second grade (ECLS-K 2010–11).

	Start of Kindergarten 1	End of Kindergarten 2	Start of First Grade 3	End of First Grade 4	Start of Second Grade 5	End of Second Grade 6

Approaches to Learning						
Low SES	−0.17	−0.13	−0.09	−0.22	−0.18	−0.26
High SES	0.32	0.28	0.30	0.36	0.30	0.36
Black	−0.12	−0.15	−0.15	−0.30	−0.11	−0.27
White	0.15	0.13	0.10	0.10	0.08	0.05
Female	0.29	0.30	0.28	0.27	0.28	0.27
Male	−0.13	−0.16	−0.18	−0.21	−0.18	−0.24
Interpersonal Skills						
Low SES	−0.13	−0.09	−0.09	−0.17	−0.15	−0.20
High SES	0.24	0.22	0.24	0.29	0.26	0.30
Black	−0.04	−0.18	−0.14	−0.26	−0.11	−0.21
White	0.13	0.13	0.15	0.10	0.12	0.07
Female	0.24	0.26	0.24	0.23	0.25	0.23
Male	−0.11	−0.13	−0.15	−0.17	−0.13	−0.20
Self-Control						
Low SES	−0.13	−0.10	−0.04	−0.17	−0.10	−0.18
High SES	0.21	0.20	0.22	0.28	0.20	0.28
Black	−0.15	−0.25	−0.18	−0.28	−0.25	−0.32
White	0.12	0.11	0.13	0.10	0.07	0.05
Female	0.23	0.21	0.22	0.20	0.21	0.19
Male	−0.12	−0.12	−0.16	−0.15	−0.13	−0.17
Externalizing Problem Behavior						
Low SES	−0.08	−0.06	−0.02	−0.10	−0.08	−0.14
High SES	0.19	0.22	0.22	0.24	0.11	0.22
Black	−0.19	−0.31	−0.23	−0.28	−0.27	−0.39
White	0.08	0.09	0.04	0.04	−0.07	0.01
Female	0.23	0.22	0.24	0.23	0.20	0.21
Male	−0.16	−0.15	−0.21	−0.19	−0.22	−0.23
Internalizing Problem Behaviors						
Low SES	−0.02	−0.05	−0.10	−0.09	−0.18	−0.13
High SES	0.10	0.18	0.12	0.15	0.12	0.18
Black	0.05	−0.03	−0.17	−0.11	−0.04	−0.09
White	0.01	0.05	0.00	−0.01	−0.02	−0.02
Female	0.06	0.06	0.00	0.04	0.06	0.05
Male	−0.01	0.01	0.05	−0.04	−0.08	−0.06

*Note*: All variables were standardized. Externalizing and internalizing problems were reverse coded so that higher values indicate better behavior. Low and high SES were defined as the top and bottom quintiles, respectively.

**Table 2:** Estimates from hierarchical linear models predicting gaps in approaches to learning at kindergarten entry and growth in gaps during school and nonschool periods (ECLS-K 2011).

	Without teacher fixed effects		With teacher fixed effects	
	Unstandardized (1)	Standardized (2)	Unstandardized (3)	Standardized (4)

Start of Kindergarten				
Female vs Male	0.315^†^	0.464^†^	0.314^†^	0.461^†^
	(0.013)	(0.018)	(0.011)	(0.015)
Black vs White	−0.090^†^	−0.130^†^	−0.169^†^	−0.251^†^
	(0.020)	(0.029)	(0.023)	(0.034)
High vs Low SES	0.320^†^	0.469^†^	0.343^†^	0.506^†^
	(0.022)	(0.021)	(0.021)	(0.031)
Kindergarten				
Female vs Male	0.002	0.003	0.002	0.002
	(0.002)	(0.003)	(0.002)	(0.002)
Black vs White	−0.007^†^	−0.010^†^	−0.006	−0.008
	(0.003)	(0.004)	(0.004)	(0.005)
High vs Low SES	−0.008^†^	−0.013^†^	−0.004	−0.006
	(0.003)	(0.004)	(0.003)	(0.005)
Summer after Kindergarten				
Female vs Male	−0.003	−0.004	−0.007	−0.009
	(0.008)	(0.011)	(0.008)	(0.011)
Black vs White	0.014	0.026	0.026	0.035
	(0.014)	(0.021)	(0.019)	(0.028)
High vs Low SES	0.012	0.024	−0.006	−0.007
	(0.014)	(0.020)	(0.016)	(0.023)
First Grade				
Female vs Male	0.002	0.002	0.005^∗^	0.006
	(0.003)	(0.004)	(0.003)	(0.004)
Black vs White	−0.007	−0.012^∗^	−0.012^∗^	−0.017^∗^
	(0.005)	(0.007)	(0.007)	(0.010)
High vs Low SES	0.010^†^	0.012^∗^	0.011^∗^	0.014^∗^
	(0.005)	(0.007)	(0.006)	(0.008)
Summer after First Grade				
Female vs Male	−0.01	−0.011	−0.003	−0.001
	(0.008)	(0.012)	(0.009)	(0.013)
Black vs White	0.028^∗^	0.043^∗^	0.025	0.034
	(0.015)	(0.022)	(0.022)	(0.032)
High vs Low SES	−0.002	0.005	−0.01	−0.01
	(0.014)	(0.021)	(0.018)	(0.026)
Second Grade				
Female vs Male	0.006^†^	0.007^∗^	0.006^∗^	0.006
	(0.003)	(0.004)	(0.003)	(0.004)
Black vs White	−0.009^∗^	−0.013^∗^	−0.001	0.000
	(0.005)	(0.007)	(0.007)	(0.011)
High vs Low SES	0.004	0.003	0.004	0.004
	(0.005)	(0.007)	(0.006)	(0.009)
Observations	55,070	55,070	55,070	55,070

Standard errors are in parentheses.

∗ *p <* 0.10,

† *p <* 0.05

**Table 3:** Planned contrasts between estimates from the three school years (kindergarten, first grade, and second grade) versus the two summers (summer after kindergarten and summer after first grade; ECLS-K 2011).

		Without teacher fixed effects		With teacher fixed effects	
Outcome	Contrast	Unstandardized (1)	Standardized (2)	Unstandardized (3)	Standardized (4)

Approaches	Female-male	0.01	0.011	0.009	0.01
		(0.008)	(0.011)	(0.008)	(0.011)
	Black-white	−0.029^†^	−0.046^†^	−0.032	−0.043
		(0.014)	(0.020)	(0.019)	(0.028)
	High SES-low SES	−0.003	−0.013	0.012	0.012
		(0.013)	(0.019)	(0.016)	(0.023)
Externalizing	Female-male	0.005	−0.001	0.003	−0.003
		(0.007)	(0.011)	(0.007)	(0.012)
	Black-white	−0.008	−0.001	−0.013	−0.012
		(0.013)	(0.020)	(0.018)	(0.028)
	High SES-low SES	0.026^†^	0.039^†^	0.008	0.01
		(0.012)	(0.019)	(0.014)	(0.023)
Internalizing	Female-male	0.003	0.007	0.014^†^	0.028
		(0.007)	(0.013)	(0.007)	(0.013)
	Black-white	−0.012	−0.035	−0.022	−0.046
		(0.012)	(0.025)	(0.017)	(0.033)
	High SES-low SES	−0.005	−0.013	−0.006	−0.011
		(0.005)	(0.024)	(0.014)	(0.027)
Interpersonal	Female-male	0.014*	0.012	0.019^†^	0.02
		(0.008)	(0.012)	(0.008)	(0.012)
	Black-white	−0.022	−0.034	−0.008	−0.004
		(0.015)	(0.023)	(0.020)	(0.030)
	High SES-low SES	−0.011	−0.03	−0.021	−0.042
		(0.014)	(0.022)	(0.016)	(0.025)
Self-Control	Female-male	−0.01	−0.022	0.002	−0.002
		(0.008)	(0.012)	(0.008)	(0.012)
	Black-white	−0.005	−0.002	0.016	0.034
		(0.014)	(0.023)	(0.019)	(0.030)
	High SES-low SES	0.006	0.004	−0.007	−0.017
		(0.014)	(0.022)	(0.016)	(0.025)

Standard errors are in parentheses.

∗ *p <* 0.10,

† *p <* 0.05

## Footnotes

1 An exception is the patterns for black and white achievement gaps, which are often observed to grow faster when school is in versus out.

2 “Above average” was defined by the distribution of teachers in terms of how well they promoted children’s improvement in an area (e.g., math, reading, and social/behavioral skills).

3 Several sampling weights are available in the ECLS-K. The sampling weight W6CF6P_2T0 was most appropriate for our analysis because it adjusts for nonresponse associated with child assessment data from both kindergarten rounds, both first-grade rounds, and both second-grade rounds as well as parent data from fall or spring kindergarten and either teacher- or child-level teacher data from spring kindergarten, spring first grade, and spring second grade (Tourangeau et al. 2017).

4 The school random effects represented the schools where the child started kindergarten. This reflected the sample design, but some children did transfer schools in later rounds. The model treated transfer students as nested in their original schools but added fixed effects for their new teachers.
