# Supplementary material for: Socioeconomic, Ethnic, Racial, and Gender Gaps in Children’s Social/Behavioral Skills: Do They Grow Faster in School or out?
Source: Sociol Sci. Author manuscript; Available in PMC 2024 Mar 8. (PMC10923568; doi:10.15195/v6.a17)
Supplement: Supp Material [file NIHMS1913825-supplement-Supp_Material.pdf]

sociological science

Supplement to:

Downey, Douglas B., Joseph Workman, and Paul von Hippel. 2019. "Socioeconomic, Ethnic, Racial, and Gender Gaps in Children's Social/Behavioral Skills: Do They Grow Faster in School or out?" Sociological Science 6: 446-466.

Table A1. Dates and reliabilities of social and behavioral assessments (ECLS-K:2010), with dates for the start and end of each school year.

		Dates		Test reliability				
		Mean	SD (days)	*Approaches to Learning*	Self-control	Interpersonal skills	Intern. Behaviors	Extern Behavior
Kindergarten	School starts	Aug 25, 2010	10					
	Fall test	Oct 10, 2010	25	0.91	0.81	0.86	0.79	0.88
	Spring test	Apr 04, 2011	21	0.91	0.82	0.87	0.78	0.89
	School ends	Jun 06, 2011	11					
1st grade	School starts	Aug 24, 2011	10					
	Fall test	Oct 10, 2011	24	0.90	0.79	0.85	0.77	0.88
	Spring test	Apr 04, 2012	21	0.91	0.81	0.86	0.76	0.86
	School ends	Jun 04, 2012	11					
2nd grade	School starts	Aug 24, 2012	9	0.91	0.80	0.85	0.78	0.88
	Fall test	Oct 10 2012	24	0.91	0.81	0.86	0.78	0.87
	Spring test	Apr 4, 2013	20					
	School ends	June 5, 2013	10					

Table B—Stata Code for Analysis

```
clear
set maxvar 20000
cd "Set Directory"

*** Recode teacher behavior variables ***
use "K-3 All Variables.dta", clear

destring T1_ID-T7_ID, replace

rename X*TCH*, lower

foreach wave in 1 2 3 4 4k 5 6 {
 foreach scale in con per ext int app {
  /* Clean up missing value codes */
  recode x`wave'tch`scale' (-9=.) (-1=.), gen(tch`scale'`wave')
 }
 /* Reverse-code the ext scale and re-standardize it. */
 replace tchext`wave'=5-tchext`wave'
}

/* Consolidate wave 4 measures */
foreach scale in con per ext int app {
 replace tch`scale'4=tch`scale'4k if missing(tch`scale'4) & !missing(tch`scale'4k)
 drop tch`scale'4k
}

/* Standardize */
foreach wave in 1 2 3 4 5 6 {
 foreach scale in con per ext int app {
  egen sttch`scale'`wave'=std(tch`scale'`wave')
 }
}

rename x*tch*, upper

* Gender (relevant to overweight) *
*Girl
recode X_CHSEX_R (2=1) (1=0) (-9=.), gen(female)

** Race/Ethnicity **
*Asian black hispanic mixed native white
recode X_RACETH_R (1=1) (2/8=0) (-9=.), gen(white)
recode X_RACETH_R (1=0) (2=1) (3/8=0) (-9=.), gen(black)
recode X_RACETH_R (1/2=0) (3/4=1) (5/8=0) (-9=.), gen(hispanic)
recode X_RACETH_R (1/4=0) (5/6=1) (7/8=0) (-9=.), gen(asian)
```

```
recode X_RACETH_R (1/6=0) (7=1) (8=0) (-9=.), gen(native)
recode X_RACETH_R (1/7=0) (8=1)(-9=.), gen(mixed)

*** SES Measure ***
 * Highest and Lowest Quintile *
 * Individual Level

recode X12SESL (-9=.), gen(SESYK)
recode X4SESL_I (-9=.), gen(SESY1)

egen SESave=rmean(SESYK SESY1)

xtile SEScat=SESave, n(5)

recode SEScat (1=1) (2/5=0), gen(ISESLOW)
recode SEScat (1=0) (2/4=1) (5=0), gen(ISESMID)
recode SEScat (1/4=0) (5=1), gen(ISESHIGH)

recode X2FLCH2_I (-9=.), gen(FLK)
recode X4FMEAL_I (-9=.), gen(FL1)

*** Age at Kindergarten Entry ***
recode X1AGEENT (-9/-1=.), gen(ageK)
 sum ageK

recode T1COMPDD T2COMPDD T3COMPDD T4COMPDD T5COMPDD
T6COMPDD T4KCOMPDD (-9/-1=.)
recode T1COMPMM T2COMPMM T3COMPMM T4COMPMM T5COMPMM
T6COMPMM T4KCOMPMM (-9/-1=.)
recode T1COMPYY T2COMPYY T3COMPYY T4COMPYY T5COMPYY T6COMPYY
T4KCOMPYY (-9/-1=.)

replace T4COMPDD=T4KCOMPDD if T4COMPDD==.
replace T4COMPMM=T4KCOMPMM if T4COMPMM==.
replace T4COMPYY=T4KCOMPYY if T4COMPYY==.
* 416-420 Changes Made *

gen Tassessdate1=mdy(T1COMPMM, T1COMPDD, T1COMPYY)
format Tassessdate1 %td
gen Tassessdate2=mdy(T2COMPMM, T2COMPDD, T2COMPYY)
format Tassessdate2 %td
gen Tassessdate3=mdy(T3COMPMM, T3COMPDD, T3COMPYY)
format Tassessdate3 %td
gen Tassessdate4=mdy(T4COMPMM, T4COMPDD, T4COMPYY)
format Tassessdate4 %td
gen Tassessdate5=mdy(T5COMPMM, T5COMPDD, T5COMPYY)
format Tassessdate5 %td
gen Tassessdate6=mdy(T6COMPMM, T6COMPDD, T6COMPYY)
```

```
format Tassessdate6 %td

sum Tassessdate*

gen DIFFTassess21=Tassessdate2-Tassessdate1
gen DIFFTassess32=Tassessdate3-Tassessdate2
gen DIFFTassess43=Tassessdate4-Tassessdate3
gen DIFFTassess54=Tassessdate5-Tassessdate4
gen DIFFTassess65=Tassessdate6-Tassessdate5

* Start and End Dates *
recode X2SCHBDD X4SCHBDD X2SCHEDD X4SCHEDD (-9/-1=.)
sum X2SCHBMM X4SCHBMM X2SCHEMM X4SCHEMM X6SCHEMM
sum X2SCHBYY X4SCHBYY X2SCHEYY X4SCHEYY X6SCHEYY
sum X2SCHBDD X4SCHBDD X2SCHEDD X4SCHEDD X6SCHEDD

recode X2SCHBMM X4SCHBMM X6SCHBMM X2SCHEMM X4SCHEMM
X6SCHEMM X2SCHBYY X4SCHBYY X6SCHBYY X2SCHEYY X4SCHEYY
X6SCHEYY X2SCHBDD X4SCHBDD X6SCHBDD X2SCHEDD X4SCHEDD
X6SCHEDD  (-9=.) (-1=.)

gen Kstartdate=mdy(X2SCHBMM, X2SCHBDD, X2SCHBYY) if X1HGTFLG==1 |
X1WGTFLG==1
format Kstartdate %td
gen Kenddate=mdy(X2SCHEMM, X2SCHEDD, X2SCHEYY) if X2HGTFLG==1 |
X2WGTFLG==1
format Kenddate %td
gen firststartdate=mdy(X4SCHBMM, X4SCHBDD, X4SCHBYY)
format firststartdate %td
gen firstenddate=mdy(X4SCHEMM, X4SCHEDD, X4SCHEYY) if X4HGTFLG==1 |
X4WGTFLG==1
format firstenddate %td
gen Secondstartdate=mdy(X6SCHBMM, X6SCHBDD, X6SCHBYY)
format Secondstartdate %td
gen Secondenddate=mdy(X6SCHEMM, X6SCHEDD, X6SCHEYY) if X6HGTFLG==1 |
X6WGTFLG==1
format Secondenddate %td
* I use the flags to only include those sample members who actually participated in a given
wave *

sum Kstartdate Kenddate firststartdate firstenddate Secondstartdate Secondenddate
*tab X2SCHBMM X2SCHBDD if Kstartdate>18480 & Kstartdate<18510
*tab X2SCHEMM X2SCHEDD if Kenddate>18775 & Kenddate<18795
*tab X4SCHBMM X4SCHBDD if firststartdate>18850 & firststartdate<18875
*tab X4SCHEMM X4SCHEDD if firstenddate>19130 & firstenddate<19155
*tab X6SCHBMM X6SCHBDD if Secondstartdate>19215 & Secondstartdate<19235
*tab X6SCHEMM X6SCHEDD if Secondenddate>19500 & Secondenddate<19525
```

```
gen DIFFstartendK=Kenddate-Kstartdate
gen DIFFstartendF=firstenddate-firststartdate
gen DIFFstartendS=Secondenddate-Secondstartdate

gen fall1sample=.
replace fall1sample=1 if X3RDGFLG==1 | X3MTHFLG==1
gen fall2sample=.
replace fall2sample=1 if X5RDGFLG==1 | X5MTHFLG==1

gen assesssummer2=.
replace assesssummer2=1 if Tassessdate2>Kenddate & Tassessdate2!=. & Kenddate!=.
* 73 changes made *
gen assesssummer3=.
replace assesssummer3=1 if Tassessdate3<firststartdate & Tassessdate3!=. &
firststartdate!=.
* 4 changes made *
gen assesssummer4=.
replace assesssummer4=1 if Tassessdate4>firstenddate & Tassessdate4!=. & firstenddate!=.
* 58 changes made *
gen assesssummer5=.
replace assesssummer5=1 if Tassessdate5<Secondstartdate & Tassessdate5!=. &
Secondstartdate!=.
* 6 changes made *
gen assesssummer6=.
replace assesssummer6=1 if Tassessdate6>Secondenddate & Tassessdate6!=. &
Secondenddate!=.
* 49 changes made *

gen monthsK1=Tassessdate1-Kstartdate
replace monthsK1=0 if Tassessdate1<Kstartdate
gen monthsK2=Tassessdate2-Kstartdate
*Problem if assessdate2 is after Kenddate
replace monthsK2=Kenddate-Kstartdate if assesssummer2==1
* 33 real changes made *
gen monthsK3=Kenddate-Kstartdate
gen monthsK4=Kenddate-Kstartdate
gen monthsK5=Kenddate-Kstartdate
gen monthsK6=Kenddate-Kstartdate

gen months1summer1=0
gen months1summer2=0
replace months1summer2=Tassessdate2-Kenddate if assesssummer2==1
* 73 changes made *
gen months1summer3=firststartdate-Kenddate if fall1sample==1
replace months1summer3=Tassessdate3-Kenddate if assesssummer3==1
* 3 changes made *
gen months1summer4=firststartdate-Kenddate if firststartdate>Kenddate
gen months1summer5=months1summer4
```

```
gen months1summer6=months1summer4

gen monthsfirst1=0
gen monthsfirst2=0
gen monthsfirst3=.
replace monthsfirst3=Tassessdate3-firststartdate if fall1sample==1 &
(Tassessdate3>firststartdate)
gen monthsfirst4=Tassessdate4-firststartdate
replace monthsfirst4=firstenddate-firststartdate if assesssummer4==1
gen monthsfirst5=firstenddate-firststartdate
gen monthsfirst6=firstenddate-firststartdate

gen months2summer1=0
gen months2summer2=0
gen months2summer3=0
gen months2summer4=0
replace months2summer4=Tassessdate4-firstenddate if assesssummer4==1
gen months2summer5=.
replace months2summer5=Secondstartdate-firstenddate if Secondstartdate>firstenddate &
fall2sample==1
replace months2summer5=Tassessdate5-firstenddate if assesssummer5==1

gen months2summer6=Secondstartdate-firstenddate if Secondstartdate>firstenddate

gen monthssecond1=0
gen monthssecond2=0
gen monthssecond3=0
gen monthssecond4=0
replace monthssecond4=Tassessdate4-Secondstartdate if Tassessdate4>Secondstartdate
gen monthssecond5=.
replace monthssecond5=Tassessdate5-Secondstartdate if fall2sample==1 &
(Tassessdate5>Secondstartdate)
gen monthssecond6=Tassessdate6-Secondstartdate
replace monthssecond6=Secondenddate-Secondstartdate if assesssummer6==1

replace monthsK1=monthsK1/30
replace monthsK2=monthsK2/30
replace monthsK3=monthsK3/30
replace monthsK4=monthsK4/30
replace monthsK5=monthsK5/30
replace monthsK6=monthsK6/30
replace months1summer1=months1summer1/30
replace months1summer2=months1summer2/30
replace months1summer3=months1summer3/30
replace months1summer4=months1summer4/30
replace months1summer5=months1summer5/30
replace months1summer6=months1summer6/30
replace monthsfirst1=monthsfirst1/30
```

```
replace monthsfirst2=monthsfirst2/30
replace monthsfirst3=monthsfirst3/30
replace monthsfirst4=monthsfirst4/30
replace monthsfirst5=monthsfirst5/30
replace monthsfirst6=monthsfirst6/30
replace months2summer1=months2summer1/30
replace months2summer2=months2summer2/30
replace months2summer3=months2summer3/30
replace months2summer4=months2summer4/30
replace months2summer5=months2summer5/30
replace months2summer6=months2summer6/30
replace monthssecond1=monthssecond1/30
replace monthssecond2=monthssecond2/30
replace monthssecond3=monthssecond3/30
replace monthssecond4=monthssecond4/30
replace monthssecond5=monthssecond5/30
replace monthssecond6=monthssecond6/30

replace monthsK3=. if fall1sample!=1
replace months1summer3=. if fall1sample!=1
replace monthsfirst3=. if fall1sample!=1
replace months2summer3=. if fall2sample!=1
replace monthssecond3=. if fall2sample!=1
replace monthsK5=. if fall2sample!=1
replace months1summer5=. if fall2sample!=1
replace monthsfirst5=. if fall2sample!=1
replace months2summer5=. if fall2sample!=1

sum months*

*** Weights ***
rename W6CF6P_2B0 fsampw
rename W6CS6P_20 ssampw

*** Need to move to long form ***
drop X* P* A* V*
drop W* IF* F* C3* R* Y* Z* S2* S4* E*
drop D* B2*
rename CHILDID, lower
drop C*
drop TWIN_ID-S7COMPYY
rename childid, upper

drop T7_ID S7_ID
forvalues wave=1/6 {
 rename T`wave'_ID T_ID`wave'
}
foreach wave in 1 3 5 6 {
```

```
 rename S`wave'_ID S_ID`wave'
}
destring S_ID1, gen(S1_ID)

reshape long S_ID T_ID tchcon tchper tchext tchint tchapp sttchcon sttchper sttchext
sttchint sttchapp assessdate monthsK months1summer monthsfirst months2summer
monthssecond, i(CHILDID) j(wave)
destring CHILDID, replace ignore("C")
destring S_ID, replace
drop Kstartdate-assesssummer6


** Running Mixed Models in Stata **
local individ female ageK black hisp asian native mixed ISESMID ISESHIGH
foreach x of local individ {
gen K`x'=monthsK*`x'
gen S1`x'=months1summer*`x'
gen F`x'=monthsfirst*`x'
gen S2`x'=months2summer*`x'
gen Sec`x'=monthssecond*`x'
}

local Kindivid Kfemale KageK Kblack Khisp Kasian Knative Kmixed KISESMID
KISESHIGH
local S1individ S1female S1ageK S1black S1hisp S1asian S1native S1mixed S1ISESMID
S1ISESHIGH
local Findivid Ffemale FageK Fblack Fhisp Fasian Fnative Fmixed FISESMID FISESHIGH
local S2individ S2female S2ageK S2black S2hisp S2asian S2native S2mixed S2ISESMID
S2ISESHIGH
local Secindivid Secfemale SecageK Secblack Sechisp Secasian Secnative Secmixed
SecISESMID SecISESHIGH
local individ female ageK black hispanic asian native mixed ISESMID ISESHIGH

mixed tchcon monthsK months1summer monthsfirst months2summer monthssecond
`individ' `Kindivid' `S1individ' `Findivid' `S2individ' `Secindivid' || CHILDID: || S1_ID: ,
emiterate(100)
estimates store conscale
lincom ((.333*Kfemale)+(.333*Ffemale)+(.333*Secfemale))-((.5*S1female)+(.5*S2female))
lincom ((.333*Kblack)+(.333*Fblack)+( .333*Secblack))-((.5*S1black)+(.5*S2black))
lincom ((.333*KISESHIGH)+(.333*FISESHIGH)+( .333*SecISESHIGH))-
((.5*S1ISESHIGH)+(.5*S2ISESHIGH))

mixed tchper  monthsK months1summer monthsfirst months2summer monthssecond
`individ' `Kindivid' `S1individ' `Findivid' `S2individ' `Secindivid' || CHILDID: || S1_ID: ,
emiterate(100)
estimates store perscale
lincom ((.333*Kfemale)+(.333*Ffemale)+(.333*Secfemale))-((.5*S1female)+(.5*S2female))
lincom ((.333*Kblack)+(.333*Fblack)+( .333*Secblack))-((.5*S1black)+(.5*S2black))
```

```
lincom ((.333*KISESHIGH)+(.333*FISESHIGH)+( .333*SecISESHIGH))-
((.5*S1ISESHIGH)+(.5*S2ISESHIGH))


mixed tchext  monthsK months1summer monthsfirst months2summer monthssecond
`individ' `Kindivid' `S1individ' `Findivid' `S2individ' `Secindivid' || CHILDID: || S1_ID: ,
emiterate(100)
estimates store extscale
lincom ((.333*Kfemale)+(.333*Ffemale)+(.333*Secfemale))-((.5*S1female)+(.5*S2female))
lincom ((.333*Kblack)+(.333*Fblack)+( .333*Secblack))-((.5*S1black)+(.5*S2black))
lincom ((.333*KISESHIGH)+(.333*FISESHIGH)+( .333*SecISESHIGH))-
((.5*S1ISESHIGH)+(.5*S2ISESHIGH))

mixed tchint  monthsK months1summer monthsfirst months2summer monthssecond
`individ' `Kindivid' `S1individ' `Findivid' `S2individ' `Secindivid' || CHILDID: || S1_ID: ,
emiterate(100)
estimates store intscale
lincom ((.333*Kfemale)+(.333*Ffemale)+(.333*Secfemale))-((.5*S1female)+(.5*S2female))
lincom ((.333*Kblack)+(.333*Fblack)+( .333*Secblack))-((.5*S1black)+(.5*S2black))
lincom ((.333*KISESHIGH)+(.333*FISESHIGH)+( .333*SecISESHIGH))-
((.5*S1ISESHIGH)+(.5*S2ISESHIGH))

mixed tchapp monthsK months1summer monthsfirst months2summer monthssecond
`individ' `Kindivid' `S1individ' `Findivid' `S2individ' `Secindivid' || CHILDID: || S1_ID: ,
emiterate(100)
estimates store appscale
lincom ((.333*Kfemale)+(.333*Ffemale)+(.333*Secfemale))-((.5*S1female)+(.5*S2female))
lincom ((.333*Kblack)+(.333*Fblack)+( .333*Secblack))-((.5*S1black)+(.5*S2black))
lincom ((.333*KISESHIGH)+(.333*FISESHIGH)+( .333*SecISESHIGH))-
((.5*S1ISESHIGH)+(.5*S2ISESHIGH))

local Kindivid Kfemale KageK Kblack Khisp Kasian Knative Kmixed KISESMID
KISESHIGH
local S1individ S1female S1ageK S1black S1hisp S1asian S1native S1mixed S1ISESMID
S1ISESHIGH
local Findivid Ffemale FageK Fblack Fhisp Fasian Fnative Fmixed FISESMID FISESHIGH
local S2individ S2female S2ageK S2black S2hisp S2asian S2native S2mixed S2ISESMID
S2ISESHIGH
local Secindivid Secfemale SecageK Secblack Sechisp Secasian Secnative Secmixed
SecISESMID SecISESHIGH
local individ female ageK black hispanic asian native mixed ISESMID ISESHIGH

mixed sttchcon monthsK months1summer monthsfirst months2summer monthssecond
`individ' `Kindivid' `S1individ' `Findivid' `S2individ' `Secindivid' || CHILDID: || S1_ID: ,
emiterate(100)
estimates store constd
lincom ((.333*Kfemale)+(.333*Ffemale)+(.333*Secfemale))-((.5*S1female)+(.5*S2female))
lincom ((.333*Kblack)+(.333*Fblack)+( .333*Secblack))-((.5*S1black)+(.5*S2black))
```

```
lincom ((.333*KISESHIGH)+(.333*FISESHIGH)+( .333*SecISESHIGH))-
((.5*S1ISESHIGH)+(.5*S2ISESHIGH))

mixed sttchper  monthsK months1summer monthsfirst months2summer monthssecond
`individ' `Kindivid' `S1individ' `Findivid' `S2individ' `Secindivid' || CHILDID: || S1_ID: ,
emiterate(100)
estimates store perstd
lincom ((.333*Kfemale)+(.333*Ffemale)+(.333*Secfemale))-((.5*S1female)+(.5*S2female))
lincom ((.333*Kblack)+(.333*Fblack)+( .333*Secblack))-((.5*S1black)+(.5*S2black))
lincom ((.333*KISESHIGH)+(.333*FISESHIGH)+( .333*SecISESHIGH))-
((.5*S1ISESHIGH)+(.5*S2ISESHIGH))

mixed sttchext  monthsK months1summer monthsfirst months2summer monthssecond
`individ' `Kindivid' `S1individ' `Findivid' `S2individ' `Secindivid' || CHILDID: || S1_ID: ,
emiterate(100)
estimates store extstd
lincom ((.333*Kfemale)+(.333*Ffemale)+(.333*Secfemale))-((.5*S1female)+(.5*S2female))
lincom ((.333*Kblack)+(.333*Fblack)+( .333*Secblack))-((.5*S1black)+(.5*S2black))
lincom ((.333*KISESHIGH)+(.333*FISESHIGH)+( .333*SecISESHIGH))-
((.5*S1ISESHIGH)+(.5*S2ISESHIGH))

mixed sttchint  monthsK months1summer monthsfirst months2summer monthssecond
`individ' `Kindivid' `S1individ' `Findivid' `S2individ' `Secindivid' || CHILDID: || S1_ID: ,
emiterate(100)
estimates store intstd
lincom ((.333*Kfemale)+(.333*Ffemale)+(.333*Secfemale))-((.5*S1female)+(.5*S2female))
lincom ((.333*Kblack)+(.333*Fblack)+( .333*Secblack))-((.5*S1black)+(.5*S2black))
lincom ((.333*KISESHIGH)+(.333*FISESHIGH)+( .333*SecISESHIGH))-
((.5*S1ISESHIGH)+(.5*S2ISESHIGH))

mixed sttchapp monthsK months1summer monthsfirst months2summer monthssecond
`individ' `Kindivid' `S1individ' `Findivid' `S2individ' `Secindivid' || CHILDID: || S1_ID: ,
emiterate(100)
estimates store appstd
lincom ((.333*Kfemale)+(.333*Ffemale)+(.333*Secfemale))-((.5*S1female)+(.5*S2female))
lincom ((.333*Kblack)+(.333*Fblack)+( .333*Secblack))-((.5*S1black)+(.5*S2black))
lincom ((.333*KISESHIGH)+(.333*FISESHIGH)+( .333*SecISESHIGH))-
((.5*S1ISESHIGH)+(.5*S2ISESHIGH))

estimates table conscale perscale extscale intscale appscale,  b(%7.2f) star(.05 .01 .001)
stats(N)
estimates table constd perstd extstd intstd appstd,  b(%7.2f) star(.05 .01 .001) stats(N)

*** Teacher-center variables
*use "Non Cog Final Data, Non-Centered.dta", clear
sort T_ID CHILDID wave

local vars_to_center tchcon tchper tchext tchint tchapp ///
```

```
        sttchcon sttchper sttchext sttchint sttchapp ///
                    female white black hispanic asian native mixed ///
                    SESave ISESLOW ISESHIGH ISESMID ageK

foreach var in `vars_to_center' {
 egen `var'_mean = mean(`var'), by(T_ID)
 gen fe`var' = `var' - `var'_mean
 drop `var'_mean
}

local individ fefemale feageK feblack fehisp feasian fenative femixed feISESMID
feISESHIGH
foreach x of local individ {
gen K`x'=monthsK*`x'
gen S1`x'=months1summer*`x'
gen F`x'=monthsfirst*`x'
gen S2`x'=months2summer*`x'
gen Sec`x'=monthssecond*`x'
}

local Kfeindivid Kfefemale KfeageK Kfeblack Kfehisp Kfeasian Kfenative Kfemixed
KfeISESMID KfeISESHIGH
local S1feindivid S1fefemale S1feageK S1feblack S1fehisp S1feasian S1fenative S1femixed
S1feISESMID S1feISESHIGH
local Ffeindivid Ffefemale FfeageK Ffeblack Ffehisp Ffeasian Ffenative Ffemixed
FfeISESMID FfeISESHIGH
local S2feindivid S2fefemale S2feageK S2feblack S2fehisp S2feasian S2fenative S2femixed
S2feISESMID S2feISESHIGH
local Secfeindivid Secfefemale SecfeageK Secfeblack Secfehisp Secfeasian Secfenative
Secfemixed SecfeISESMID SecfeISESHIGH
local feindivid fefemale feageK feblack fehisp feasian fenative femixed feISESMID
feISESHIGH

mixed fetchcon monthsK months1summer monthsfirst months2summer monthssecond
`feindivid' `Kfeindivid' `S1feindivid' `Ffeindivid' `S2feindivid' `Secfeindivid' || CHILDID:
|| S1_ID: , emiterate(100)
estimates store confe
lincom ((.333*Kfefemale)+(.333*Ffefemale)+(.333*Secfefemale))-
((.5*S1fefemale)+(.5*S2fefemale))
lincom ((.333*Kfeblack)+(.333*Ffeblack)+( .333*Secfeblack))-
((.5*S1feblack)+(.5*S2feblack))
lincom ((.333*KfeISESHIGH)+(.333*FfeISESHIGH)+( .333*SecfeISESHIGH))-
((.5*S1feISESHIGH)+(.5*S2feISESHIGH))

mixed fetchper  monthsK months1summer monthsfirst months2summer monthssecond
`feindivid' `Kfeindivid' `S1feindivid' `Ffeindivid' `S2feindivid' `Secfeindivid' || CHILDID:
|| S1_ID: , emiterate(100)
estimates store perfe
```

```
lincom ((.333*Kfefemale)+(.333*Ffefemale)+(.333*Secfefemale))-
((.5*S1fefemale)+(.5*S2fefemale))
lincom ((.333*Kfeblack)+(.333*Ffeblack)+( .333*Secfeblack))-
((.5*S1feblack)+(.5*S2feblack))
lincom ((.333*KfeISESHIGH)+(.333*FfeISESHIGH)+( .333*SecfeISESHIGH))-
((.5*S1feISESHIGH)+(.5*S2feISESHIGH))

mixed fetchext  monthsK months1summer monthsfirst months2summer monthssecond
`feindivid' `Kfeindivid' `S1feindivid' `Ffeindivid' `S2feindivid' `Secfeindivid' || CHILDID:
|| S1_ID: , emiterate(100)
estimates store extfe
lincom ((.333*Kfefemale)+(.333*Ffefemale)+(.333*Secfefemale))-
((.5*S1fefemale)+(.5*S2fefemale))
lincom ((.333*Kfeblack)+(.333*Ffeblack)+( .333*Secfeblack))-
((.5*S1feblack)+(.5*S2feblack))
lincom ((.333*KfeISESHIGH)+(.333*FfeISESHIGH)+( .333*SecfeISESHIGH))-
((.5*S1feISESHIGH)+(.5*S2feISESHIGH))

mixed fetchint  monthsK months1summer monthsfirst months2summer monthssecond
`feindivid' `Kfeindivid' `S1feindivid' `Ffeindivid' `S2feindivid' `Secfeindivid' || CHILDID:
|| S1_ID: , emiterate(100)
estimates store intfe
lincom ((.333*Kfefemale)+(.333*Ffefemale)+(.333*Secfefemale))-
((.5*S1fefemale)+(.5*S2fefemale))
lincom ((.333*Kfeblack)+(.333*Ffeblack)+( .333*Secfeblack))-
((.5*S1feblack)+(.5*S2feblack))
lincom ((.333*KfeISESHIGH)+(.333*FfeISESHIGH)+( .333*SecfeISESHIGH))-
((.5*S1feISESHIGH)+(.5*S2feISESHIGH))

mixed fetchapp monthsK months1summer monthsfirst months2summer monthssecond
`feindivid' `Kfeindivid' `S1feindivid' `Ffeindivid' `S2feindivid' `Secfeindivid' || CHILDID:
|| S1_ID: , emiterate(100)
estimates store appfe
lincom ((.333*Kfefemale)+(.333*Ffefemale)+(.333*Secfefemale))-
((.5*S1fefemale)+(.5*S2fefemale))
lincom ((.333*Kfeblack)+(.333*Ffeblack)+( .333*Secfeblack))-
((.5*S1feblack)+(.5*S2feblack))
lincom ((.333*KfeISESHIGH)+(.333*FfeISESHIGH)+( .333*SecfeISESHIGH))-
((.5*S1feISESHIGH)+(.5*S2feISESHIGH))

mixed festtchcon monthsK months1summer monthsfirst months2summer monthssecond
`feindivid' `Kfeindivid' `S1feindivid' `Ffeindivid' `S2feindivid' `Secfeindivid' || CHILDID:
|| S1_ID: , emiterate(100)
estimates store confestd
lincom ((.333*Kfefemale)+(.333*Ffefemale)+(.333*Secfefemale))-
((.5*S1fefemale)+(.5*S2fefemale))
lincom ((.333*Kfeblack)+(.333*Ffeblack)+( .333*Secfeblack))-
((.5*S1feblack)+(.5*S2feblack))
```

```
lincom ((.333*KfeISESHIGH)+(.333*FfeISESHIGH)+( .333*SecfeISESHIGH))-
((.5*S1feISESHIGH)+(.5*S2feISESHIGH))

mixed festtchper  monthsK months1summer monthsfirst months2summer monthssecond
`feindivid' `Kfeindivid' `S1feindivid' `Ffeindivid' `S2feindivid' `Secfeindivid' || CHILDID:
|| S1_ID: , emiterate(100)
estimates store perfestd
lincom ((.333*Kfefemale)+(.333*Ffefemale)+(.333*Secfefemale))-
((.5*S1fefemale)+(.5*S2fefemale))
lincom ((.333*Kfeblack)+(.333*Ffeblack)+( .333*Secfeblack))-
((.5*S1feblack)+(.5*S2feblack))
lincom ((.333*KfeISESHIGH)+(.333*FfeISESHIGH)+( .333*SecfeISESHIGH))-
((.5*S1feISESHIGH)+(.5*S2feISESHIGH))

mixed festtchext  monthsK months1summer monthsfirst months2summer monthssecond
`feindivid' `Kfeindivid' `S1feindivid' `Ffeindivid' `S2feindivid' `Secfeindivid' || CHILDID:
|| S1_ID: , emiterate(100)
estimates store extfestd
lincom ((.333*Kfefemale)+(.333*Ffefemale)+(.333*Secfefemale))-
((.5*S1fefemale)+(.5*S2fefemale))
lincom ((.333*Kfeblack)+(.333*Ffeblack)+( .333*Secfeblack))-
((.5*S1feblack)+(.5*S2feblack))
lincom ((.333*KfeISESHIGH)+(.333*FfeISESHIGH)+( .333*SecfeISESHIGH))-
((.5*S1feISESHIGH)+(.5*S2feISESHIGH))

mixed festtchint  monthsK months1summer monthsfirst months2summer monthssecond
`feindivid' `Kfeindivid' `S1feindivid' `Ffeindivid' `S2feindivid' `Secfeindivid' || CHILDID:
|| S1_ID: , emiterate(100)
estimates store intfestd
lincom ((.333*Kfefemale)+(.333*Ffefemale)+(.333*Secfefemale))-
((.5*S1fefemale)+(.5*S2fefemale))
lincom ((.333*Kfeblack)+(.333*Ffeblack)+( .333*Secfeblack))-
((.5*S1feblack)+(.5*S2feblack))
lincom ((.333*KfeISESHIGH)+(.333*FfeISESHIGH)+( .333*SecfeISESHIGH))-
((.5*S1feISESHIGH)+(.5*S2feISESHIGH))

mixed festtchapp monthsK months1summer monthsfirst months2summer monthssecond
`feindivid' `Kfeindivid' `S1feindivid' `Ffeindivid' `S2feindivid' `Secfeindivid' || CHILDID:
|| S1_ID: , emiterate(100)
estimates store appfestd
lincom ((.333*Kfefemale)+(.333*Ffefemale)+(.333*Secfefemale))-
((.5*S1fefemale)+(.5*S2fefemale))
lincom ((.333*Kfeblack)+(.333*Ffeblack)+( .333*Secfeblack))-
((.5*S1feblack)+(.5*S2feblack))
lincom ((.333*KfeISESHIGH)+(.333*FfeISESHIGH)+( .333*SecfeISESHIGH))-
((.5*S1feISESHIGH)+(.5*S2feISESHIGH))

estimates table confe perfe extfe intfe appfe,  b(%7.2f) star(.05 .01 .001) stats(N)
```

```
estimates table confestd perfestd extfestd intfestd appfestd,  b(%7.2f) star(.05 .01 .001)
stats(N)
```

Estimates From Hierarchical Linear Models Predicting Gaps in Self-Control, Interpersonal Skills, Externalizing Problem Behaviors, and Internalizing Problem Behaviors at Kindergarten Entry and Growth in Gaps During School and Non-School Periods.

Table C1--Self Control

	Without teacher FE				With teacher FE			
	Unstandardized		Standardized		Unstandardized		Standardized	
	Est	SE	Est	SE	Est	SE	Est	SE
Beginning of Kindergarten								
Female vs Male	0.241*	(0.012)	0.384*	(0.020)	0.221*	(0.011)	0.353*	(0.017)
Black vs White	-0.116*	(0.020)	-0.185*	(0.032)	-0.164*	(0.023)	-0.263*	(0.037)
Hi vs Low SES	0.203*	(0.021)	0.324*	(0.034)	0.178*	(0.021)	0.286*	(0.034)
Kindergarten								
Female vs Male	-0.003	(0.002)	-0.005+	(0.003)	-0.001	(0.002)	-0.002	(0.003)
Black vs White	-0.010*	(0.003)	-0.015*	(0.005)	-0.005	(0.004)	-0.007	(0.006)
Hi vs Low SES	-0.005+	(0.003)	-0.009+	(0.005)	-0.001	(0.003)	-0.003	(0.005)
Summer after Kindergarten								
Female vs Male	0.009	(0.008)	0.020	(0.013)	-0.001	(0.008)	0.003	(0.013)
Black vs White	0.001	(0.015)	-0.003	(0.024)	0.004	(0.019)	0.000	(0.031)
Hi vs Low SES	-0.009	(0.014)	-0.009	(0.023)	-0.006	(0.016)	-0.006	(0.026)
First Grade								
Female vs Male	-0.002	(0.003)	-0.003	(0.004)	0.002	(0.003)	0.004	(0.004)
Black vs White	0.001	(0.005)	0.001	(0.008)	-0.001	(0.007)	-0.000	(0.011)
Hi vs Low SES	0.013*	(0.005)	0.020*	(0.008)	0.004	(0.006)	0.006	(0.009)
Summer after First Grade								
Female vs Male	0.007	(0.009)	0.016	(0.014)	-0.002	(0.009)	0.002	(0.014)
Black vs White	0.001	(0.016)	-0.003	(0.025)	-0.030	(0.022)	-0.055	(0.034)
Hi vs Low SES	0.001	(0.015)	0.006	(0.024)	0.018	(0.018)	0.034	(0.029)
Second Grade								
Female vs Male	-0.000	(0.003)	-0.003	(0.005)	0.002	(0.003)	0.000	(0.005)
Black vs White	-0.002	(0.005)	-0.002	(0.008)	0.014+	(0.007)	0.025*	(0.012)
Hi vs Low SES	-0.001	(0.005)	-0.003	(0.008)	-0.006	(0.006)	-0.011	(0.010)

+ p<0.10, * p<0.05. Analyses based upon 52,921 observations; 14,219 students; 838 schools

Table C2--Interpersonal Skills

	Without teacher FE				With teacher FE			
	Unstandardized		Standardized		Unstandardized		Standardized	
	Est	SE	Est	SE	Est	SE	Est	SE
Beginning of Kindergarten								
Female vs Male	0.241*	(0.013)	0.379*	(0.020)	0.234*	(0.011)	0.369*	(0.017)
Black vs White	-0.067*	(0.020)	-0.105*	(0.031)	-0.158*	(0.024)	-0.249*	(0.036)
Hi vs Low SES	0.231*	(0.022)	0.361*	(0.034)	0.228*	(0.022)	0.361*	(0.033)
Kindergarten								
Female vs Male	0.003	(0.002)	0.003	(0.003)	0.003	(0.002)	0.003	(0.003)
Black vs White	-0.011*	(0.003)	-0.016*	(0.005)	-0.008+	(0.004)	-0.010+	(0.006)
Hi vs Low SES	-0.008*	(0.003)	-0.014*	(0.005)	-0.005	(0.004)	-0.009	(0.005)
Summer after Kindergarten								
Female vs Male	-0.007	(0.008)	-0.005	(0.013)	-0.014+	(0.008)	-0.016	(0.013)
Black vs White	0.005	(0.015)	0.009	(0.024)	0.005	(0.020)	0.000	(0.030)
Hi vs Low SES	0.012	(0.015)	0.029	(0.023)	0.010	(0.017)	0.022	(0.026)
First Grade								
Female vs Male	0.004	(0.003)	0.004	(0.004)	0.007*	(0.003)	0.008+	(0.004)
Black vs White	-0.002	(0.005)	-0.004	(0.008)	-0.001	(0.007)	0.001	(0.011)
Hi vs Low SES	0.009+	(0.005)	0.011	(0.008)	0.003	(0.006)	0.003	(0.009)
Summer after First Grade								
Female vs Male	-0.012	(0.009)	-0.011	(0.014)	-0.012	(0.009)	-0.011	(0.014)
Black vs White	0.024	(0.017)	0.036	(0.026)	0.007	(0.022)	0.005	(0.035)
Hi vs Low SES	0.009	(0.016)	0.025	(0.024)	0.025	(0.019)	0.047	(0.029)
Second Grade								
Female vs Male	0.006+	(0.003)	0.006	(0.005)	0.007*	(0.003)	0.008	(0.005)
Black vs White	-0.010+	(0.006)	-0.015+	(0.009)	0.003	(0.008)	0.006	(0.012)
Hi vs Low SES	-0.001	(0.005)	-0.005	(0.008)	-0.009	(0.007)	-0.017+	(0.010)

+ p<0.10, * p<0.05. Analyses based upon 53,242 observations; 14,234 students; 838 schools

Table C3--Externalizing Problem Behaviors

	Without teacher FE				With teacher FE			
	Unstandardized		Standardized		Unstandardized		Standardized	
	Est	SE	Est	SE	Est	SE	Est	SE
Beginning of Kindergarten								
Female vs Male	0.273*	(0.012)	0.437*	(0.018)	0.261*	(0.010)	0.418*	(0.016)
Black vs White	-0.139*	(0.018)	-0.223*	(0.030)	-0.149*	(0.022)	-0.238*	(0.035)
Hi vs Low SES	0.144*	(0.020)	0.231*	(0.032)	0.134*	(0.020)	0.214*	(0.032)
Kindergarten								
Female vs Male	-0.002	(0.002)	-0.005*	(0.003)	-0.002	(0.002)	-0.004	(0.003)
Black vs White	-0.006*	(0.003)	-0.009*	(0.004)	-0.008*	(0.004)	-0.011*	(0.006)
Hi vs Low SES	0.003	(0.003)	0.004	(0.004)	0.001	(0.003)	0.001	(0.005)
Summer after Kindergarten								
Female vs Male	0.001	(0.007)	0.008	(0.011)	0.004	(0.007)	0.014	(0.012)
Black vs White	0.003	(0.013)	-0.005	(0.021)	0.014	(0.018)	0.017	(0.028)
Hi vs Low SES	-0.029*	(0.012)	-0.043*	(0.020)	-0.025+	(0.015)	-0.037	(0.024)
First Grade								
Female vs Male	0.002	(0.002)	0.003	(0.004)	0.001	(0.003)	0.002	(0.004)
Black vs White	0.001	(0.004)	0.004	(0.007)	-0.003	(0.006)	-0.005	(0.010)
Hi vs Low SES	0.011*	(0.004)	0.019*	(0.007)	0.007	(0.005)	0.011	(0.008)
Summer after First Grade								
Female vs Male	-0.008	(0.007)	-0.005	(0.012)	-0.008	(0.008)	-0.005	(0.013)
Black vs White	0.005	(0.014)	-0.004	(0.022)	0.003	(0.020)	-0.003	(0.032)
Hi vs Low SES	-0.012	(0.013)	-0.018	(0.021)	0.012	(0.017)	0.023	(0.027)
Second Grade								
Female vs Male	0.005+	(0.002)	0.005	(0.004)	0.004	(0.003)	0.004	(0.004)
Black vs White	-0.008+	(0.005)	-0.011	(0.007)	-0.002	(0.007)	-0.001	(0.011)
Hi vs Low SES	0.002	(0.004)	0.002	(0.007)	-0.002	(0.006)	-0.004	(0.009)

+ p<0.10, * p<0.05. Analyses based upon 54,497 observations; 14,285 students; 838 schools

Table C4--Internalizing Problem Behaviors

	Without teacher FE				With teacher FE			
	Unstandardized		Standardized		Unstandardized		Standardized	
	Est	SE	Est	SE	Est	SE	Est	SE
Beginning of Kindergarten								
Female vs Male	-0.042*	(0.010)	-0.085*	(0.020)	-0.038*	(0.009)	-0.078*	(0.018)
Black vs White	-0.041*	(0.016)	-0.081*	(0.032)	-0.003	(0.019)	-0.006	(0.039)
Hi vs Low SES	-0.083*	(0.017)	-0.169*	(0.035)	-0.111*	(0.018)	-0.226*	(0.035)
Kindergarten								
Female vs Male	0.001	(0.002)	0.003	(0.003)	0.001	(0.001)	0.002	(0.003)
Black vs White	0.006*	(0.003)	0.012*	(0.005)	0.009*	(0.003)	0.017*	(0.007)
Hi vs Low SES	-0.004	(0.003)	-0.009	(0.006)	-0.000	(0.003)	-0.000	(0.006)
Summer after Kindergarten								
Female vs Male	-0.003	(0.007)	-0.006	(0.014)	-0.011	(0.007)	-0.022	(0.014)
Black vs White	-0.005	(0.013)	-0.001	(0.026)	0.007	(0.017)	0.015	(0.033)
Hi vs Low SES	0.005	(0.012)	0.014	(0.025)	0.004	(0.014)	0.008	(0.028)
First Grade								
Female vs Male	-0.001	(0.002)	-0.002	(0.005)	0.003	(0.002)	0.006	(0.005)
Black vs White	-0.001	(0.004)	-0.006	(0.009)	-0.005	(0.006)	-0.010	(0.012)
Hi vs Low SES	-0.006	(0.004)	-0.012	(0.008)	-0.003	(0.005)	-0.005	(0.010)
Summer after First Grade								
Female vs Male	-0.003	(0.008)	-0.008	(0.015)	-0.013	(0.008)	-0.026+	(0.015)
Black vs White	0.027*	(0.014)	0.064*	(0.028)	0.033+	(0.019)	0.069+	(0.038)
Hi vs Low SES	-0.002	(0.013)	-0.002	(0.026)	0.003	(0.016)	0.006	(0.032)
Second Grade								
Female vs Male	-0.001	(0.003)	-0.000	(0.005)	0.002	(0.003)	0.005	(0.005)
Black vs White	-0.008	(0.005)	-0.016+	(0.009)	-0.009	(0.007)	-0.018	(0.013)
Hi vs Low SES	-0.001	(0.005)	-0.001	(0.009)	-0.004	(0.006)	-0.008	(0.011)

+ p<0.10, * p<0.05. Analyses based upon 54,086 observations; 14,271 students; 838 schools
